# Expression of the EGF Family in Gastric Cancer: Downregulation of HER4 and Its Activating Ligand NRG4

**DOI:** 10.1371/journal.pone.0094606

**Published:** 2014-04-11

**Authors:** Trine Ostergaard Nielsen, Lennart Friis-Hansen, Steen Seier Poulsen, Birgitte Federspiel, Boe Sandahl Sorensen

**Affiliations:** 1 Department of Clinical Biochemistry, Aarhus University Hospital, Aarhus C, Denmark; 2 Department of Biomedical Sciences, and The Novo Nordisk Foundation Center for Basic Metabolic Research, Faculty of Health and Medical Sciences, University of Copenhagen, Copenhagen, Denmark; 3 Center for Genomic Medicine, Rigshospitalet, University of Copenhagen, Rigshospitalet, Copenhagen, Denmark; 4 Department of Pathology, Copenhagen University Hospital, Rigshospitalet, Copenhagen, Denmark; Vanderbilt University School of Medicine, United States of America

## Abstract

Gastric cancer is a major cause of cancer-related deaths in both men and women. The epidermal growth factor receptors are EGFR, HER2, HER3 and HER4. Of the four epidermal growth factor receptors, EGFR and HER2 are well-known oncogenes involved in gastric cancer. Little, however, is known about the role played by HER3 and HER4 in this disease. We obtained paired samples from the tumor and the adjacent normal tissue from the same patient undergoing surgery for gastric cancer. Using RT-qPCR, we quantified the mRNA expression of the four receptors including the HER4 splicing isoforms and all the ligands activating these receptors. Using immunohistochemistry, the protein expression of HER4 was also quantified. We found that HER2 mRNA expression was upregulated in the tumor tissue compared to the matched normal tissue (p = 0.0520). All ligands with affinity for EGFR were upregulated, whereas the expression of EGFR was unchanged. Interestingly, we found the mRNA expression of HER4 (p = 0.0002) and its ligand NRG4 (p = 0.0009) to be downregulated in the tumor tissue compared to the matched normal tissue. HER4 downregulation was demonstrated for all the alternatively spliced isoforms of this receptor. These results support the involvement of EGFR and HER2 in gastric cancer and suggest an interesting association of reduced HER4 expression with development of gastric cancer.

## Introduction

Gastric cancer is the third leading cause of cancer-related deaths in men and the fifth leading cause in women. Gastric cancer accounted for approximately 990,000 new registered cases and 738,000 deaths worldwide in 2008 [1]. Infection with *Helicobacter pylori*, diet, smoking and alcohol consumption are major determinants influencing the risk of getting gastric cancer [2], [3].

The epidermal growth factor (EGF) family is a complex network of tyrosine kinase receptors and ligands interacting to induce various signaling cascades. The family includes the four receptors EGFR (HER1), HER2 (ErbB2), HER3 (ErbB3), and HER4 (ErbB4) [4]. By alternative splicing, HER4 is expressed in a number of isoforms. In the juxtamembraneous area of HER4, inclusion of either exon 15b or exon 16 results in the JMa or JMb isoforms, respectively [5]–[7]. The JMa isoform and not the JMb isoform can be processed to release a soluble HER4 intracellular domain (ICD) that localizes in the cytoplasm, nucleus, and mitochondria [8], [9]. Alternative splicing in the cytoplasmic region of HER4 gives rise to the CYT1 and CYT2 isoforms. Skipping of exon 26 results in a receptor (CYT2) lacking 16 of the amino acids of the full-length receptor (CYT1) [10].

The ligands of the EGF family are epidermal growth factor (EGF), transforming growth factor-α (TGF-α), and amphiregulin (AR) that are specific for EGFR; heparin-binding EGF-like growth factor (HB-EGF), betacellulin (BTC), and epiregulin (EPR) that bind both EGFR and HER4; neuregulin 1 (NRG1) and NRG2 that bind both HER3 and HER4; and finally NRG3 and NRG4 that are specific for HER4 [11]. When binding an activating ligand, the receptors make homo- and hetero-dimers. The composition of dimerization partners and activating ligands determines the signal to be transduced inside the cell [12], [13]. The EGFR family plays various roles in the growth and maintenance of numerous tissues, among others by regulating cell division, apoptosis, differentiation, and migration. Deregulation of signaling from this system is connected to development and growth of cancers [14]–[17].

EGFR and HER2 are well-known oncogenes in gastric cancer [18], [19]. The HER2 inhibiting antibody trastuzumab successfully improves prognosis when added to the chemotherapy regimen in patients with HER2-positive advanced gastric cancers [20]. A number of clinical trials have also been conducted to test the usability of EGFR inhibitors in gastric cancer [21], [22]. HER3 and HER4 could potentially also be important in the etiology of gastric cancer; however, information about their roles in this disease is sparse. Indeed, little is known about the expression pattern of the complete EGF system. Because of the interactive nature of the EGF receptors and ligands, clarification of the mechanisms of regulation of the EGF family members demands considering all the receptors and their ligands together.

To investigate the expression pattern of the EGF receptors and ligands in gastric cancer, we obtained tissue from the tumor and the adjacent normal tissue from the same patient undergoing surgery for gastric cancer. We used paired tissue samples to ensure against the inter-individual variation that exists in the baseline expression of the EGF system. Using RT-qPCR, we quantified the mRNA expression of the four EGF receptors including the HER4 splicing isoforms and the ligands activating these receptors.

## Materials and Methods

### Patient selection and collection of biopsies

In this cohort study, 38 patients undergoing surgery for adenocarcinoma of the stomach, esophagus or gastroesophageal junction were consecutively included from July 2008 to November 2009. All individuals provided an informed written consent. Collection of biopsies was approved by the regional scientific ethics committees of Zealand, Denmark (journal number H-B-2008-049), and the establishment of a biobank was approved by the Danish Data Protection Agency (journal number 2008-41-2138). Biopsies were taken from both tumor tissue and the adjacent normal tissue. All biopsy samples were reviewed by an experienced pathologist to validate the diagnosis. Pre-defined clinical data were extracted from medical charts and pathology reports. The clinical characteristics of the patients are given in Table 1.

**Table 1:** Clinical characteristics of the patients included in this study.

	Number of patients
Total number of patients	38
**Sex**	
Female	11
Male	27
Age at diagnosis	
Median	52 years
Range	36–90 years
**Localization**	
Gastroesophageal junction	18
Antrum	13
Fundus	2
Corpus	2
Fundus and corpus	1
Unknown	2

### RNA extraction

Biopsies were placed in RNA*later* (Life Technologies, Carlsbad, CA, USA) immediately after removal. RNA*later* was removed prior to RNA isolation, and Trizol (Life Technologies, Carlsbad, CA, USA) was added. The tissue samples were homogenized on a Tissuelyser (Qiagen, Chatsworth, CA, USA). RNA from tissue was isolated according to the manufacture's protocol for Trizol RNA isolation (Life Technologies, Carlsbad, CA, USA). RNA concentrations were measured on a NanoDrop ND-1000 Spectrophotometer. RNA quality was tested on a 2100 Bioanalyzer (Agilent Technologies, Santa Clara, CA, USA) according to the manufacture's protocol.

### Reverse transcription quantitative polymerase chain reaction (RT-q-PCR)

cDNA was synthesized from 1 ug total RNA using High Capacity cDNA Reverse Transcription Kit (Life Technologies, Carlsbad, CA, USA). The final reaction was diluted to a total volume of 200 uL.

1 µl of cDNA was used for qPCR using SYBR green Master mix (Roche, Basel, Switzerland) on a Roche lightcycler 480 at: 95°C, 10 min; 50 cycles of 95°C for 10 sec, specific annealing temperature for 10 sec, 72°C for 5 sec; 99°C for 1 sec; 59°C for 15 sec; 95°C for 1 sec; cooling to 40°C. Primer sequences and annealing temperatures are given in Table 2.

**Table 2:** Primers and annealing temperature for the qPCR reactions used in the study.

Assay	Forward primer	Reverse primer	Annealing temperature
**EGFR**	5′-GAG AAC GCC TCC CTC A-3′	5′-GGT ACT CGT CGG CAT C-3′	54°C
**HER2**	5′-CCA GGA CCT GCT GAA CTG GT-3′	5′-TGT ACG AGC CGC ACA TCC-3′	59°C
HER2 probe	Fam 5′-CAG ATT GCC AGG GGG ATG AGC TAC CTG-3′ Tamra		-
**HER3**	5′-GGT GCT GGG CTT GCT TTT-3′	5′-CGT GGC TGG AGT TGG TGT TA-3′	65°C
**HER4 total**	5′-ACA GCA GTA CCG AGC CTT TGC G-3′	5′-GCC ACT AAC ACG TAG CCT GTG AC-3′	64°C
**HER4 CYT1**	5′-GGA TGA AGA GGA TTT GGA AG-3′	5′-TCCTGACATGGGGGTGTA-3′	56°C
**HER4 CYT2**	5′-GAA TAG GAA CCA GTT TGT ATA CCG-3′	5′- ACA GCAG GAG TCA TCA AAA ATC-3′	56°C
**HER4 JMa**	5′-TAA CGG TCC CAC TAG TCA-3′	5′-CAT GTT GTG GTA AAG TGG-3′	60°C
**HER4 JMb**	5′- ATA GGC TCA AGT ATT GAA G-3′	3′-CCA TCA GGC CGA TGC-3′	60°C
**EGF**	5′-GAC TTG GGA GCC TGA GCA GAA-3′	5′-CAT GCA CAA GCG TGA CTG GAG GT-3′	66°C
**HB-EGF**	5′-GGT GGT GCT GAA GCT CTT TC-3′	5′-CCC CTT GCC TTT CTT CTT TC-3′	61°C
**AR**	5′-GCC TCA GGC CAT TAT GC-3′	5′-ACC TGT TCA ACT CTG ACT GA-3′	58°C
**BTC**	5′-TCT AGG TGC CCC AAG C-3′	5′-GTG CAG ACA CCG ATG A-3′	66°C
**EPR**	5′-AAA GTG TAG CTC TGA CAT G-3′	5′-CTG TAC CAT CTG CAG AAA TA-3′	60°C
**TGF-α**	5′-GCC CGC CCG TAA AAT GGT CCC CTC-3′	5′-GTC CAC CTG GCC AAA CTC CTC CTC TGG G-3′	70°C
**NRG1α**	5′-ATC CAC CAC TGG GAC A-3′	5′-TTT GGA TCA TGG GCA-3′	60°C
**NRG1β**	5′-TAG GAA ATG ACA GTG CCT C-3′	5′-CGT AGT TTT GGC AGC GA-3′	65°C
**NRG2α**	5′- AAA TAT GGC AAC GGC AG-3′	5′-CGC AAA GGC AGT TTC T-3′	60°C
**NRG2β**	5′-GCT TTA CGT CAA CAG CG-3′	5′-CCG GTG TAT CCC ACA G-3′	63°C
**NRG3**	5′-ACA GTG CAA GCG AAA AC-3′	5′-CAC TAT GAT ATG AGG GCG-3′	61°C
**NRG4**	5′-CTG TTG TCT GCG GTA TCC-3′	5′-TCA TTC TTG GTC AAG AGA GT-3′	61°C
**SDHA**	5′-TGG GAA CAA GAG GGC ATC TG-3′	5′-CCA CCA CTG CAT CAA ATT CAT G	62°C

The gene best suited for normalization was determined by applying the Normfinder algorithm [23] to the quantified concentrations of GAPDH, β-actin, YWHAZ, SDHA, and HMBS. SDHA turned out to be the best of the five. Two samples were excluded from the analyses due to no detection of mRNA from any of the reference genes.

### Immunostaining

Paraffin-embedded tissues fixed in 4% buffered paraformaldehyde from 13 representative patients were investigated by immunohistochemistry using c-erbB-4/HER-4, rabbit polyclonal antibody diluted 1/50 as the primary antibody (RB-9045-P1, Thermo Scientific, Wilmington, DE, USA). For antigen retrieval sections were boiled in micro wave oven in 10 mM citrate buffer, pH 6.0. The immunoreaction was visualized by biotinylated goat anti-rabbit antibody diluted 1/200 (BA-1000, Vector Laboratories, Burlingame, CA, USA), followed by a preformed avidin and biotinylated horseradish peroxidase macromolecular complex (ABC) diluted 1/100 (Code nr. PK-4000, Vector Laboratories, Burlingame, CA, USA). The reaction was developed by the use of 3,3 -diaminobenzidine and counterstaining was performed with Mayer's Hemalum. As a negative control, the same procedure was conducted without any primary antibody.

Using Image Pro Plus (MediaCybernetics) the staining intensity of representative images of the tumor versus the surrounding mucosa tissue was estimated. Two images of tumor sample and two images of the surrounding mucosa were collected from each tissue sample and the average of the two values was calculated and used for statistical analysis and plotting. For a few samples, only tumor tissue or only normal mucosa was present and no comparison of HER4 expression was possible for these tissues. Paired t-test was used to determine if the intensity of the tumor tissues was different from the intensity of the mucosa tissues.

## Results

### mRNA expression of the EGF receptors in gastric cancer

**Figure 1:**
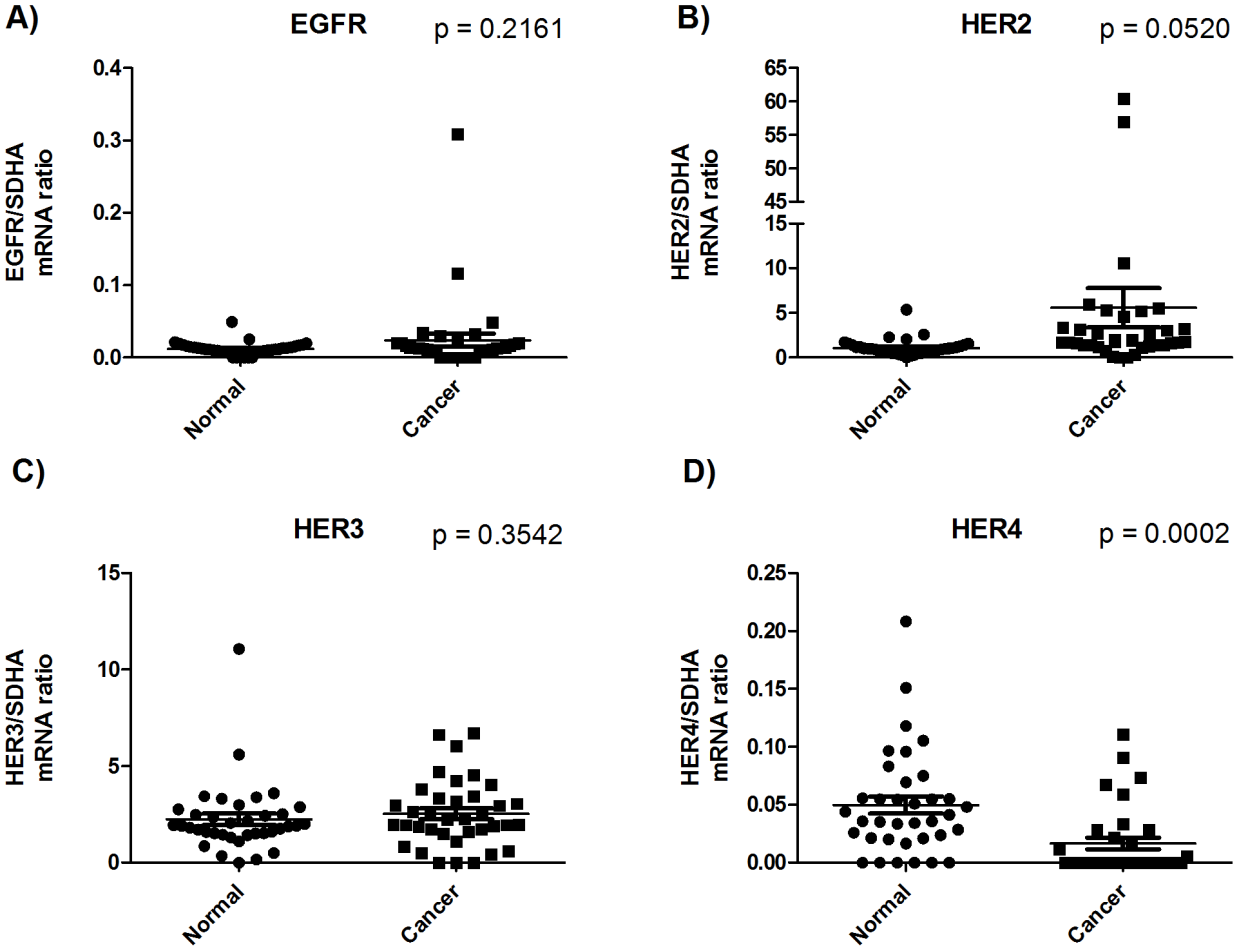
mRNA expressions of the four receptors of the EGF system. All mRNA expression concentrations are arbitrary and represent the ratio of mRNA expression of the target gene divided by the mRNA expression of the reference gene SDHA. mRNA expression was quantified in tumor biopsies from gastric cancer patients and the paired adjacent normal tissue from the same patient. P values are calculated using a paired t-test.

RNA extracted from 38 tumors and matched samples from the adjacent normal tissue from patients who underwent surgery for gastric cancer was used for RT-qPCR quantifications. The mRNA expression of the four EGF family receptors EGFR, HER2, HER3, and HER4 is depicted in Figure 1. Interestingly, the HER4 receptor was subjected to downregulation in the cancerous tissue compared to the normal tissue (p = 0.0004). For HER2, we saw an upregulation of this receptor in the cancerous tissue that was very close to being significant (p = 0.0520). EGFR and HER3 mRNA expression was un-changed in the tumors compared to the normal tissue.

**Figure 2:**
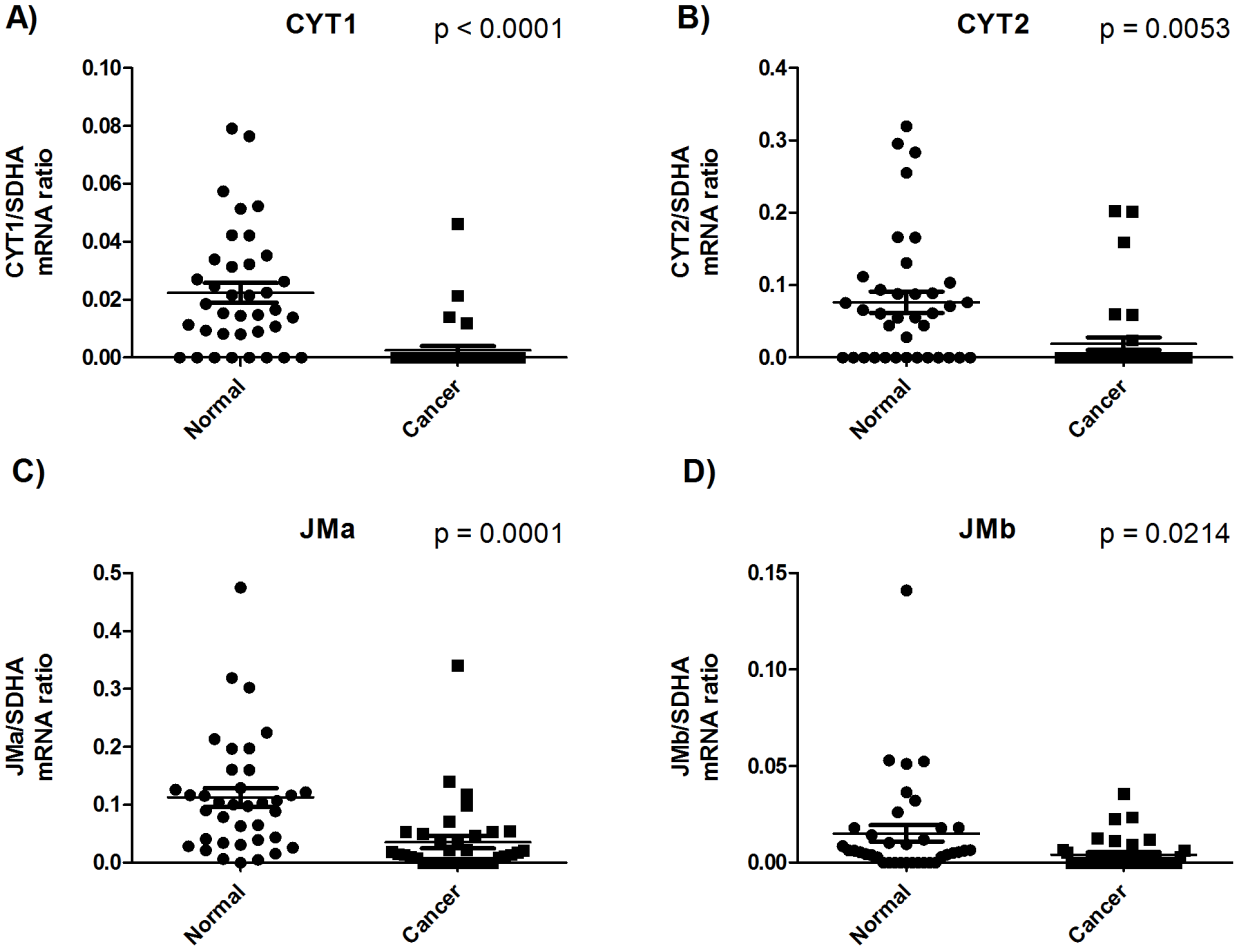
mRNA expressions of the alternatively spliced isoforms of HER4. All mRNA expression concentrations are arbitrary and represent the ratio of mRNA expression of the target gene divided by the mRNA expression of the reference gene SDHA. mRNA expression was quantified in tumor biopsies from gastric cancer patients and the paired adjacent normal tissue from the same patient. P values are calculated using a paired t-test.

HER4 exists as different alternatively spliced isoforms. The juxtamembranous region can consist of either JMa or JMb, and the cytoplasmic region can consist of either CYT1 or CYT2. The downregulation observed for total HER4 mRNA expression in cancerous tissue applied to all four variants of the receptor (Figure 2).

**Figure 3:**
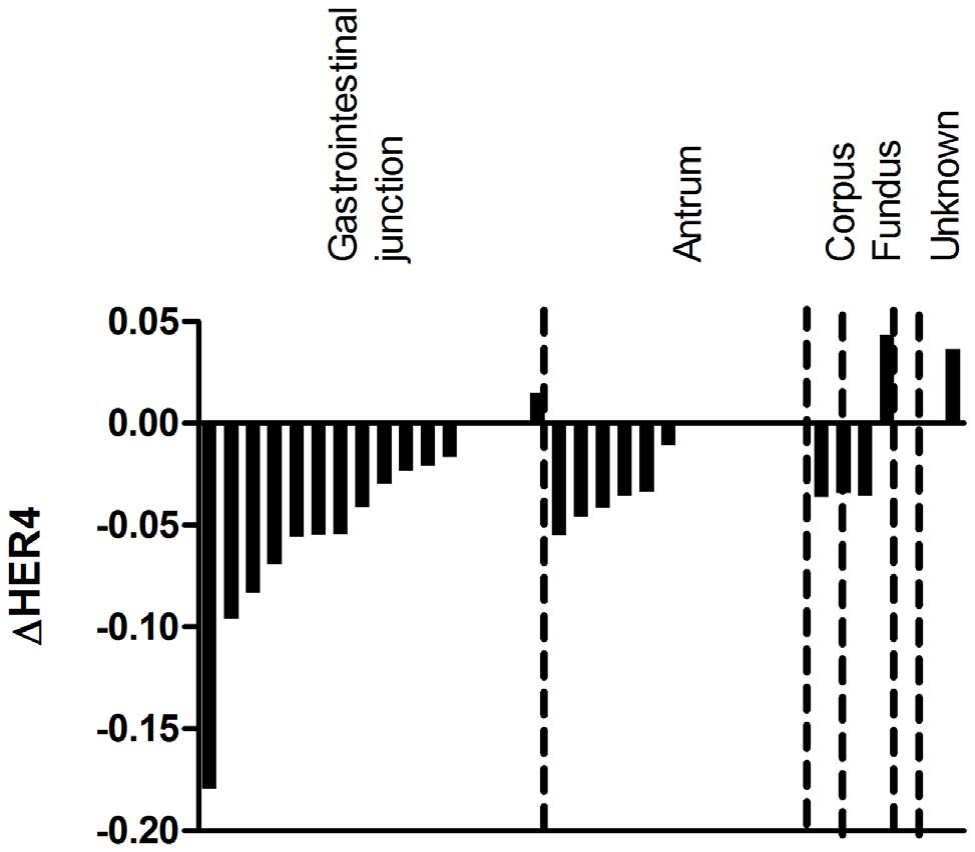
ΔHER4 mRNA expression for each patient. Waterfall plot depicting the difference in total HER4 mRNA expression between the tumor tissue and the normal tissue for each patient (ΔHER4). The difference is calculated as the tumor tissue expression minus the normal tissue expression, and hence, a negative value represents a downregulation in the tumor tissue compared to the normal tissue. The tumor localization of the individual patients is depicted in the figure.

To better illustrate the magnitude of HER4 downregulation in the tumor tissue, we plotted the HER4 down- or upregulation for each individual patient. The HER4 mRNA expression in the tumor tissue minus the HER4 mRNA expression in the matched normal tissue (ΔHER4) is depicted in Figure 3. A value below 0 represents a downregulation. This figure clearly illustrates that HER4 downregulation is a very general mechanism that takes place in the majority of the patients and regardless of tumor localization.

### mRNA expression of the EGF family ligands in gastric cancer

**Figure 4:**
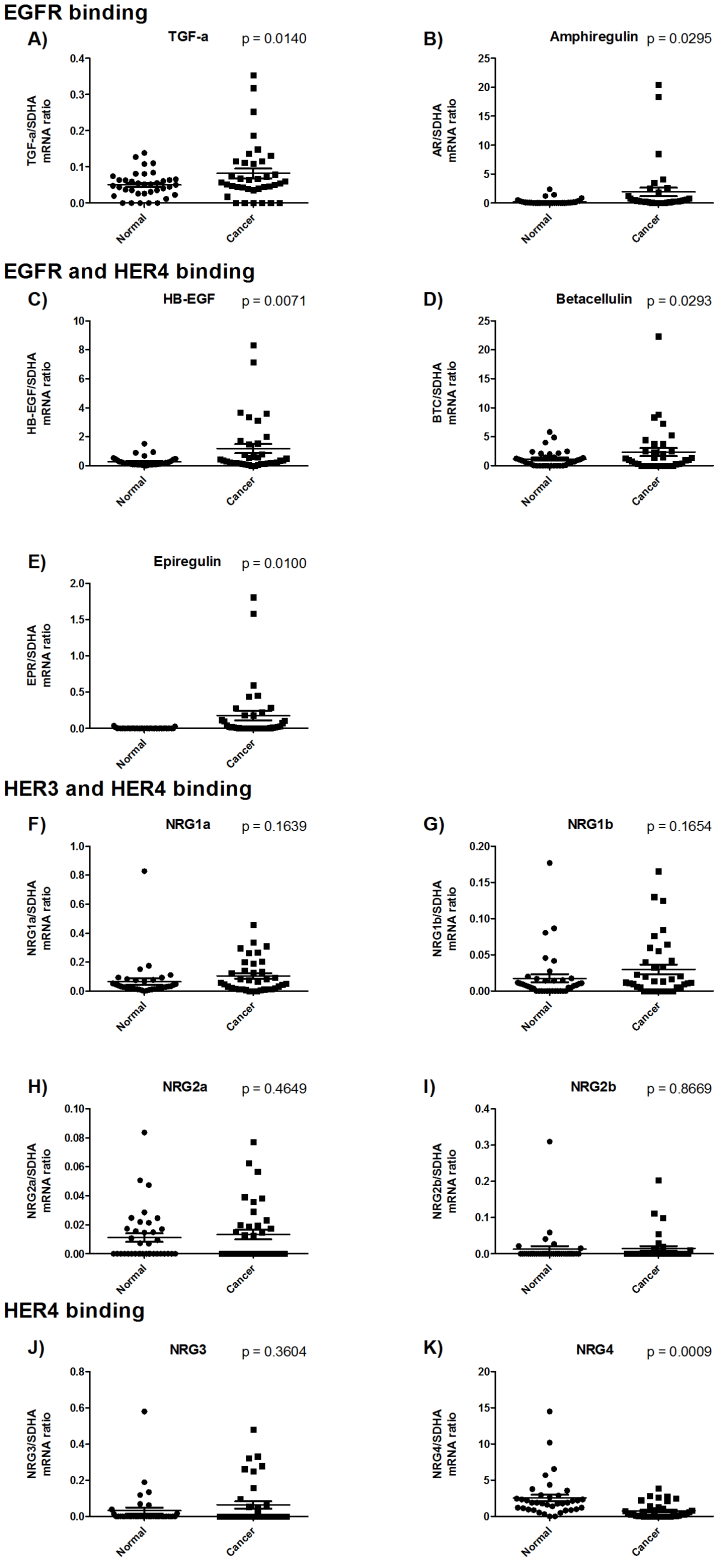
mRNA expressions of the ligands of the EGF system. For NRG1 and NRG2 the alternatively spliced variants α and β are included. All mRNA expression concentrations are arbitrary and represent the ratio of mRNA expression of the target gene divided by the mRNA expression of the reference gene SDHA. mRNA expression was quantified in tumor biopsies from gastric cancer patients and the paired adjacent normal tissue from the same patient. P values are calculated using a paired t-test.

TGF-α, amphiregulin, and EGF specifically bind EGFR. A negligible mRNA expression level was detected for EGF in both tumor and normal tissue, and therefore this ligand was not considered for further analysis. TGF-α and amphiregulin were both upregulated in the cancerous tissue compared to the normal tissue (Figures 4a and b). HB-EGF, betacellulin, and epiregulin have affinity for both EGFR and HER4, and all of these ligands were also upregulated in the cancerous tissue (Figures 4 c, d, and e). Taken together, this shows that all EGFR-activating ligands are upregulated in the tumors.

Of the neuregulins, NRG1 and NRG2 bind both HER3 and HER4. We observed no change in expression of either the α or the β variants of NRG1 and NRG2 (Figures 4f, g, h and i). NRG3 and NRG4 only bind HER4. NRG3 did not undergo any change in expression from normal to cancerous tissue (Figure 4j). However, NRG4 was downregulated in the cancerous tissue compared to the normal tissue (p = 0.0009) (Figure 4k). This demonstrates that both HER4 and its specific ligand NRG4 are down-regulated in gastric cancer.

**Figure 5:**
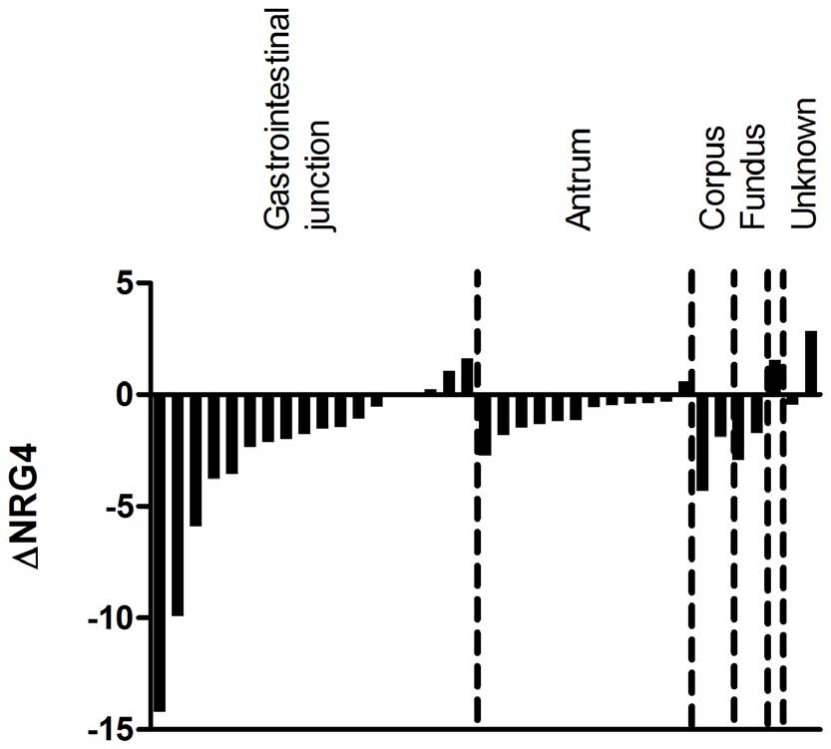
ΔNRG4 mRNA expression for each patient. Waterfall plot depicting the difference in NRG4 mRNA expression between the tumor tissue and the normal tissue for each patient (ΔNRG4). The difference is calculated as the tumor tissue expression minus the normal tissue expression, and hence, a negative value represents a downregulation in the tumor tissue compared to the normal tissue. The tumor localization of the individual patients is depicted in the figure.

To better illustrate the magnitude of NRG4 downregulation in the tumor tissue, we plotted the NRG4 down- or upregulation for each individual patient. The NRG4 mRNA expression in the tumor tissue minus the NRG4 mRNA expression in the matched normal tissue (ΔNRG4) is depicted in Figure 5.

Considering all the patients showing downregulation of total HER4 in their tumor tissue, in 82% of these NRG4 was also downregulation and in 86% one or more of the EGFR-shared ligands (HB-EGF, epiregulin, betacellulin) was upregulated. In 36% of the patients with total HER4 downregulation, all three of the EGFR shared ligands were upregulated.

### Protein expression of HER by immunohistochemistry

Because of post-transcriptional regulations, the mRNA expression patterns determined by RT-qPCR might not represent the protein expression pattern. To ensure that the observed decrease in HER4 mRNA expression in the cancerous tissue of patients with gastric cancer is also reflected on the protein level, we performed immunohistochemical staining on formalin-fixed paraffin-embedded tissue from all patients included in the study.

**Figure 6:**
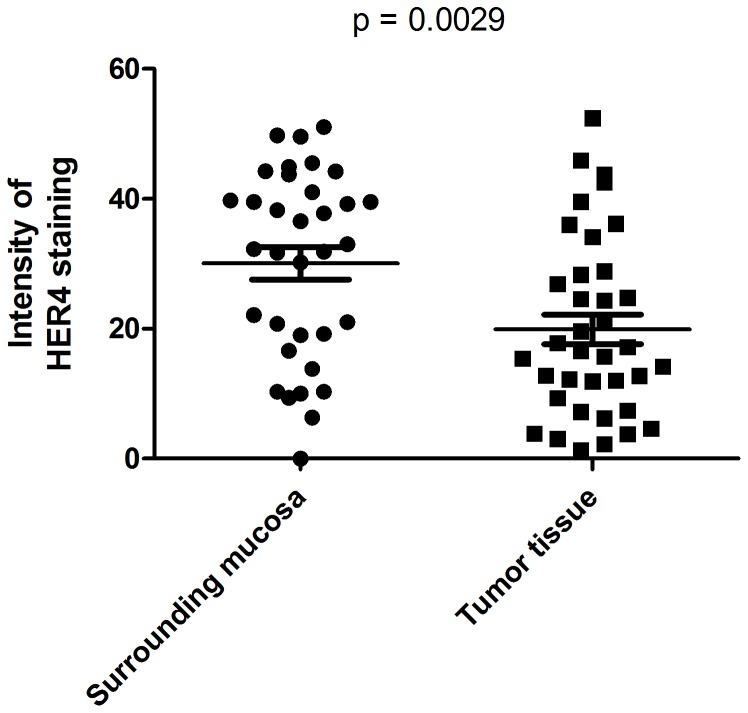
Quantification of HER4 immunostaining intensity in tumor tissue and normal tissue. The staining intensity of representative images of the tumor versus the surrounding mucosa tissue was estimated. Two images of tumor sample and two images of the normal mucosa were collected from each tissue sample and the average of the two values was calculated. P value was calculated using a paired t-test.

By a quantification of the HER4 staining intensity in representative areas of the tumor- and the surrounding tissue, we found the tumor tissue to have significantly less HER4 staining intensity compared to the surrounding normal mucosa (p = 0.0029) (Figure 6).

**Figure 7:**
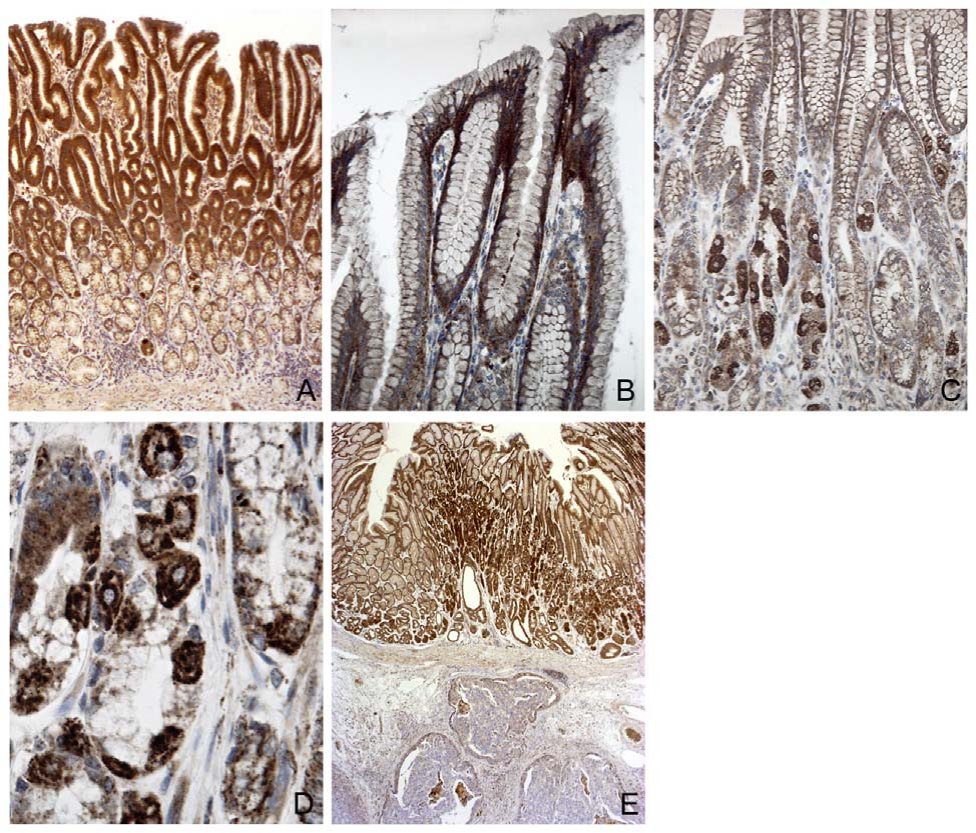
Immunohistochemical staining for HER4 protein expression in normal and cancer tissue. **A**) Normal gastric mucosa with immunoreaction to HER4 primarily in the surface epithelium but also in the glands. **B**) larger magnification of the gastric epithelium. The immunoreaction is localized basolaterally in the surface epithelial cells – both as a cytoplasmatic and membrane staining. **C**) In the glands the most intense staining was seen in the parietal cells. These (**D**)) were easily identified by the presence of intracellular cannaliculi. **E**) The normal mucosa with strong immunoreactions in the upper part of the figure contrasts with underlying negative tumor tissue. Magnifications **A**×40, **B**,**G**×210, **C**×130, **D**×600; **E**×20.

Representative photomicrographs of the stainings are presented in Figure 7. In the normal gastric mucosa there was a strong HER4 immunoreaction (Figure 7 a). Especially the basal part of the cytoplasm and the lateral cell membrane of the surface epithelial cells were strongly immunoreactive for HER4 (Figure 5 b). Also the parietal cells showed very strong HER4 immunoreactivity (Figure 7 c and d). The submucosa and muscle layers were negative. In the glandular structures especially the parietal cells showed very strong HER4 immunoreactivity (Figures 7 c and d). Negative controls performed without any primary antibody resulted in no staining.

In the gastric cancers only minor areas with tumor cells had a positive immunoreaction to HER4, and the negative tumor tissue contrasted strongly with the stained normal mucosa adjacent to the tumor (Figure 7). In the central parts of the tumor tissue almost all cells were completely negative apart from small clusters of cells which might have had a distinct membrane staining.

## Discussion

The EGF family is composed of an interacting network of receptors and ligands that must be considered as a whole to understand the regulation of signaling in the EGF family. We determined the regulation pattern of the EGF family in tumor tissue from patients with gastric cancer as well as the matched adjacent normal tissue from the same patients.

We found the signaling potential of EGFR to be upregulated in the tumor tissue through upregulation of all the EGFR activating ligands, and we also found upregulation of HER2 in tumor tissue that was very close to being significant (p = 0.0520). This is in good agreement with existing data that show that both EGFR and HER2 are associated with poor prognosis in gastric cancer [19]. The importance of HER2 in gastric cancer is demonstrated by the fact that the HER2 inhibiting antibody Herceptin is the only approved agent for targeted treatment of gastric cancer [24].

For HER3, we did not observe any expressional regulation on the mRNA level. However, a few studies on HER3 in gastric cancer have ascribed a role for HER3 in gastric cancer. HER3 has been associated with poor survival, and positive HER3 protein expression was detected by immunohistochemistry in 18.6% of gastric cancer samples compared to only 2.0% of nontumorous samples [25]–[27]. We did not find a higher HER3 expression in gastric cancer compared to normal tissue in our study. However, a difference in the methods used in the studies (immunohistochemistry vs RT-qPCR) might account for some of the discrepancies in the results. In addition, we were able to describe the regulation pattern in each patient rather than in a pool of cancer patients and a pool of control persons, and this could also account for a difference in the results obtained in the two studies.

We found a marked downregulation of both HER4 and its activating ligand NRG4 in tumor tissue. Our results are strengthened by the fact that we also found a downregulation when we used additional assays to quantify the specific isoforms of HER4. Using immunohistochemistry, we confirmed that normal gastric tissue is positive for HER4, whereas cancerous tissue has very low or no HER4 staining.

The decreased expression of HER4 mRNA expression in the tumor tissue can be ascribed to a decrease in the number of cells expressing HER4 or to a decrease in HER4 expression in all the HER4 expressing cells. From our immunohistochemical stainings, we observe that in the gastric cancers only minor clusters with tumor cells had a positive immunoreaction to HER4, whereas the vast majority of tumor cells were completely negative. This indicates that it is the number of cell that expresses HER4 that is decreased in the tumors. In contrast to the tumor tissue, the normal tissue had very strong immunoreactions to HER4. Interestingly, it is especially mucus-producing cells and hydrochloric acid-producing parietal cell that express HER4 protein.

The role of HER4 in cancerous disease is controversial. In bladder cancer, RT-qPCR quantification of HER4 and its ligands show that HER4 is associated with better survival. In particular, the co-expression of HER4 together with its specific ligand, NRG4, is highly correlated with better survival [28]. Likewise, HER4 is associated with better overall and disease-free survival in cervix cancer [29]. In laryngeal squamous cell carcinoma, HER4 is lost in late stage tumors [30], and HER4 is mainly found in low-stage and non-metastatic xenograft tumors in pancreatic cancer [31].

On the other hand, HER4 protein expression as determined by immunohistochemistry has been associated with distal metastasis and a decreased survival rate in oral squamous cell carcinomas [32]. In a study of colorectal cancer in which the protein expression pattern was also determined by immunohistochemistry, early-stage patients had a lower percentage of HER4 overexpression than late-stage patients [33]. In malignancies of the central nervous system, HER4 also possesses a tumor-promoting role [34], [35].

Not much is known about the role of HER4 in gastric cancer. No HER4 mRNA expression was detected in two gastric cancer cell lines [36]. HER4 protein expression as detected by immunohistochemistry has been found to correlate with early stage, lower grade, and absence of lymph node metastases in gastric cancer [25]. This is in very good agreement with another study that investigated gene expression profiles in long-term survivors from metastatic gastric cancer treated with chemotherapy in which the authors indicated a possible role of HER4 upregulation in these patients [37]. Together with our present results, these data further indicate that HER4 signaling has a protective function against carcinogenesis in the gastric region. One study found the HER4 gene to be frequently amplified in gastric cancer [38]. However, the results of our studies indicate that this does not translate to the HER4 expression level since we found a downregulation of HER4 mRNA and protein expression rather than an upregulation.

Even though we did not find the expression of EGFR to be regulated, the signaling potential through this receptor is increased by the upregulation of all its ligands. This includes the ligands shared with HER4: HB-EGF, betacellulin, and epiregulin. In addition to autocrine EGFR activation, upregulation of these ligands potentially results in increased activity of HER4. Activation of the EGFR pathway is indeed a way for the cells to become increasingly carcinogenic. However, considering HER4 to have anti-carcinogenic functions, a concomitant activation of this receptor would halt the proliferative advantage of EGFR activation. One could speculate that this potential activation of HER4 is avoided by the concomitant downregulation of the receptor and its specific ligand NRG4.

In patients with bladder cancer, the combined HER4/NRG4 signaling unit can be growth inhibitory, and the co-expression of HER4 together with NRG4 is highly correlated to better survival if the two proteins act together rather than individually [28].

We have also performed an analysis to compare the HER4 expression to patient survival. We did observe a trend towards high HER4 expression being correlated to a better survival (results not shown), however, due to a limitation in the number of patients included in the study, this result was not found to be significant and further studies will have to elucidate if a correlation between HER4 expression and survival can be found in gastric cancer. A correlation of the expression level of the EGF receptors to the presence of lymph node metastases and the tumor invasiveness showed only HER2 to correlate inversely to these parameters whereas no correlation was found for the remaining EGF family (results not shown).

In conclusion, our results suggest a new role for HER4 in gastric cancer. Interestingly, we found both HER4 and its specific ligand NRG4 to be downregulated in cancerous tissue. This is accompanied by an increased signaling potential of EGFR through upregulation of all its ligands and an upregulation of HER2.
